# Campbell title registrations to date – January 2021

**DOI:** 10.1002/cl2.1145

**Published:** 2021-03-18

Details of new titles for systematic reviews or evidence and gap maps that have been accepted by the Editor of a Campbell Coordinating Group are published in each issue of the journal. If you would like to receive a copy of the approved title registration form, please send an email to the Managing Editor of the relevant Coordinating Group.

## 1 CRIME AND JUSTICE

*Risk factors, mitigating factors and interventions against intergenerational transmission of extremism within family contexts: A systematic review*

Daan Weggemans, Layla van Wieringen, Katharina Krüsselmann, Tessa Ubels and Marieke Liem

5 October 2020

*The treatment of gender in crime and justice systematic reviews: An evidence and gap map*

Jieyun Li, Howard White, Liping, Guo, Jingwen Li, Na Zhang, Xiuxia Li, Kehu Yang, Guanghua Liu

16 October 2020

*Interventions to improve outcomes for children who offend: An evidence and gap map*

Howard White, Ashwani Verma, Kusha Verma, Sabina Singh, Anagha Joshi, Ashima Mohan, Jane Lewis, Hannah Gaffney and Ashrita Saran

6 December 2020

*Scientific knowledge and approaches to defining and measuring hate crime, hate speech and hate incidents: A systematic review and qualitative synthesis*

Matteo Vergani, Barbara Perry, Joshua Freilich, Steven Chermak, Ryan Scrivens

15 January 2021

## 2 EDUCATION

*Paired Reading to improve educational outcomes in children aged 4 to 11: A systematic review*

Jennifer Roberts, Paul Connolly, Judy Sebba, Laura Neeson, Sharon Millen

18 November 2020

*A systematic review and meta‐analysis of 5E‐based interventions for improving STEM outcomes*

Joshua R. Polanin, Megan Austin, Joseph Taylor, Ryan Williams

12 January 2021

## 3 INTERNATIONAL DEVELOPMENT

*The effect of digital payments for social safety nets on take‐up and transaction costs for implementers and beneficiaries in low‐ and middle‐income countries: A systematic review*

Iffat Zahan, Howard White, Ashrita Saran, Shamel Ahmed

19 October 2020

*Aquaculture for improving livelihoods, nutrition, and female empowerment in low‐ and middle‐income countries: A systematic review*

Constanza Gonzalez Parrao, Marta Moratti, Shannon Shisler, Birte Snilstveit, John Eyers

29 October 2020

*Teacher professional learning programs on the inclusion of students with disabilities in low and middle‐income countries in the Asia‐Pacific: An evidence and gap map*

Syeda Kashfee Ahmed, David Jeffries, Anannya Chakraborty, Petra Lietz, Amit Kaushik, Budiarti Rahayu, Kris Sundarsagar

8 December 2020

*Strengthening women's empowerment and gender equality in fragile contexts: A systematic review*

Etienne Lwamba, Will Ridlehoover, Meital Kupfer, Shannon Shisler, Ada Sonnenfeld, Laurenz Langer, John Eyers, Sean Grant, Bidisha Barooah

15 December 2020

*Residential energy efficiency interventions: An effectiveness systematic review*

Miriam Berretta, Joshua Furgeson, Collins Zamawe, Ian Hamilton, Yue Wu, Paul J. Ferraro, Neal Haddaway, John Eyers

6 January 2021

## 4 KNOWLEDGE TRANSLATION & IMPLEMENTATION

*Determinants of COVID health‐related behaviours (COHeRe): A rapid review*

Jennifer Hanratty, Sarah Miller, Declan Bradley, Martin Dempster

9 October 2020

## 5 SOCIAL WELFARE

*The state of knowledge on adult transracial and intercountry adoptees: An evidence and gap map*

Hollee McGinnis, Anne Farina, Amanda Baden, Kristen Kremer, Mary O'Leary Wiley

20 October 2020

*Social and behaviour change communication (SBCC) interventions for strengthening HIV prevention and research among adolescent girls and young women (AGYW) in low‐ and middle‐income countries*

Devi Leena Bose, Jessy Joseph, Kriti Goyal, Saif Ul Hadi, Ashrita Saran

20 October 2020

*The prevalence of complex trauma and its impact on at‐risk youths’ lives: A systematic review and meta‐analysis*

Patricia Correia‐Santos, Bárbara Sousa, Diogo Morgado, Ricardo J. Pinto, Julian D. Ford, Luís Azevedo, Marta Pinto, Ângela Costa Maia

10 November 2020

*The effectiveness of interventions for depression: A map of systematic reviews*

Jingwen Li, Liangying Hou, Jieyun Li, Yuanchang Jin, Xiuxia Li, Howard White, Na Zhang, Kangle Guo, Liping Guo, Lufang Feng, Xiajing Chu, Nan Chen, Kehu Yang

12 November 2020

*Shared living arrangements after divorce and the wellbeing of children: A systematic review*

Na Zhang, Xiuxia Li, Lufang Feng, Kangle Guo, Jieyun Li, Jingwen Li, Nan Chen, Kehu Yang, Xingrong Liu

16 November 2020

*Group‐based community interventions to support the social reintegration of marginalized adults with mental illness*

Karl Fritjof Krassel, Maya Christiane Flensborg Jensen, Bjørn, Christian Arleth Viinholt, Nina Thorup Dalgaard, Trine Filges

18 November 2020

*The effectiveness of home‐based interventions for child neglect: A components‐based systematic review*

Rui Li, Meixuan Li, Zhitong Bing, Xiuxia Li, Kehu Yang

26 November 2020

